# Tribute to the Greatest Mentor of All Time: Prof. Mirko Tos (1931-2018)

**DOI:** 10.4274/balkanmedj.2018.2.0001

**Published:** 2018-03-15

**Authors:** Cem Uzun

**Affiliations:** 1 Vice-Rector, responsible for the Scientific Journals of Trakya University; Department of Otolaryngology, Trakya University School of Medicine, Edirne, Turkey

Professor Mirko Tos, the best otorhinolaryngologist in the World, passed away on January 16, 2018, in Denmark at the age of 87. His books are the leading textbooks used to teach otologic surgery. He published nearly 700 articles and was the most productive otolaryngologist at the peak of his career (1).

Prof. Tos was my mentor; I was probably his last student before he retired in 2001. In addition, he has taught not only my Danish colleagues but also many others from all over the world, especially from the Balkans. We have been his ambassadors forwarding his concepts of treatment to our patients. I took the blessings of my patients and whispered them to him during his funeral in Copenhagen on January 27, 2001. May his soul rest in peace!

Prof. Mirko Tos, besides having many invitations from several countries, also visited Trakya University and Edirne several times and gave important lectures here. His first visit was in 2001, again in 2006, and his final trip was in 2010. He was the guest of honor and the invited speaker at all three meetings: the 2^nd^ Trakya Otorhinolaryngology and Head-Neck Surgery (ORL&HNS) Days-New Advances in Otology Symposium, the 5^th^ Balkan Congress of ORL&HNS, and the Trakya Society of ORL&HNS Meeting on Secretory Otitis Media, respectively. He was always happy for being in Edirne and never refused us (Picture).

Prof. Tos was originally from Slovenia and moved to Denmark in 1957 (2). He became a pioneer of otology and neurotology in Denmark and organized many important international meetings besides being the author of his famous books. After his retirement from Copenhagen University Hospital of Gentofte in 2001, he was appointed to a “first professor” position at the University of Maribor in Slovenia in 2002 until 2011 (3).

In addition to his well-known textbooks, he has several surgical techniques for the treatment of ear diseases, classifications, and theories for otologic pathologies. His classification of cartilage tympanoplasties was first published in 2007 in his article published in the Trakya University Medical Faculty Journal, currently entitled, the *Balkan Medical Journal* (4). Additionally, he served as a member of the International Advisory Board at our journal between 2005 and 2016. His contribution to our journal was also very essential during the journal’s evaluation process for the Science Citation Index Expanded (SCIE). His participation enabled the fulfillment of one of the election criteria that examines the number of both articles and citations of editorial board members and authors in that process (5).

One of the memorable mottos of Prof. Tos is that “speak depending on your own data!” (6). He often advised and asked about “research.” Every patient was a new puzzle for him; thus, he individualized the treatment of each case. These two concepts-evidence-based and personalized medicine-are relatively new approaches to the modern treatment of diseases, which I learned from him 17 years ago.

Denmark was the best country in the world regarding the number of articles published in SCIE per number of inhabitants during the end of the 90s (7). This status was mainly due to Prof. Tos’ and his co-authors’ publications (1). He never stopped, always asked new questions, and gave us ideas to search for; i.e., most of these questions he mentioned at the end of his article, which was published at our journal (4), have not been answered yet.

Prof. Tos has also been the Honorary Member of the Balkan Society of ORL&HNS and the Trakya Society of ORL&HNS. We have shared many memories, which are not forgettable (8). He has been “the Idol in Otolaryngology” and will always be our guide in our practice, with his principles and manuals.

## Figures and Tables

**Picture:**
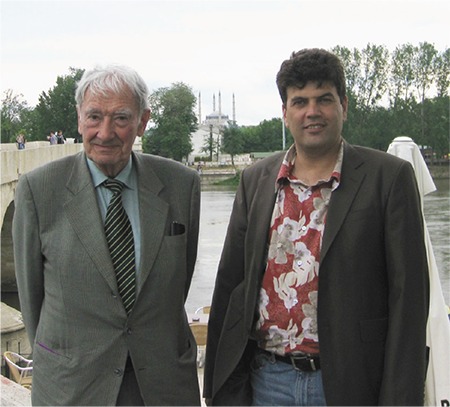
Together with Prof. Mirko Tos (left) in riverside Meric, Edirne (2010)

## References

[1] Uzun C, Tos M (2001). An Idol in Otorhinolaryngology. Pro Otology (Balkan Journal of Otology & Neurotology).

[2] Tos N (2017). The Way to Denmark from Childhood in Slovenia (Danish).

[3] Cayé-Thomasen P. In Memoriam: Mirko Tos (2018).

[4] Tos M (2007). Need For Clinical Research in Cartilage Tympanoplasty. Balkan Med J.

[5] (Updated 18 July 2016. Access: 18 February 2018). Testa J. Journal Selection Process - Clarivate.

[6] Uzun C (2018). Obituary. Professor Mirko Tos, 1931-2018. ENT & Audiology News.

[7] Stendahl O, Nilsson J (1999). Svensk medicinsk forskning tappar mark. Vårdens kvalitet hotas. MFR informerar.

[8] (Accessed: 18 February 2018). Uzun C. Prof. Mirko TOS: You have always been our idol! Global Otology Online Discussion Forum executed by Politzer Society & European Academy of Otology-Neurotology.

